# Anti-*Helicobacter pylori* activity of potential probiotic *Lactiplantibacillus pentosus *SLC13

**DOI:** 10.1186/s12866-022-02701-z

**Published:** 2022-11-21

**Authors:** Tran Thi Dieu Thuy, Pei-Yun Kuo, Sha-Ma Lin, Cheng-Yen Kao

**Affiliations:** 1 Institute of Microbiology and Immunology, College of Life Sciences, National Yang Ming Chiao Tung University, No.155, Sec.2, Linong Street, Taipei, 112 Taiwan; 2 Grace Christian Academy, Taipei, Taiwan

**Keywords:** Adhesion, Exopolysaccharide, *Helicobacter pylori*, *Lactiplantibacillus* *pentosus*, Probiotic, Urease

## Abstract

**Background:**

Here, we aimed to evaluate and compare the anti-*Helicobacter pylori* activity of potential probiotic *Lactiplantibacillus* *pentosus* SLC13 to *Lactobacillus gasseri* BCRC 14619 T and *Lacticaseibacillus rhamnosus* LGG. Phenotypic assays including growth curve, cell adhesion, and cellular cytotoxicity were performed to characterize SLC13. Anti-*H. pylori* activity of lactobacilli was determined by the disk diffusion method and co-culture assay. Exopolysaccharide (EPS) was extracted from lactobacilli to test its immune modulation activity, and IL-8 expression in AGS and GES-1 was determined by RT-qPCR.

**Results:**

All three lactobacilli strains were tolerant to the simulated gastrointestinal conditions. SLC13 showed the highest adhesion ability to AGS and GES-1 cells, compared to LGG and BCRC 14619 T. The coculture assays of SLC13, LGG, and BCRC 14619 T with cells for 4 h showed no significant cytotoxic effects on cells. All tested strains exhibited an inhibitory effect against *H. pylori* J99. The cell-free supernatant (CFS) of three strains showed activity to inhibit *H. pylori* urease activity in a dose-dependent manner and the CFS of SLC13 had the highest urease inhibitory activity, compared to LGG and BCRC 14619 T. Only the treatment of AGS cells with SLC13 EPS significantly decreased the IL-8 expression induced by *H. pylori* infection as compared to cells treated with LGG and BCRC 14619 T EPS.

**Conclusions:**

SLC13 possesses potent antimicrobial activity against *H. pylori* growth, infection, and *H. pylori-*induced inflammation. These results suggest that SLC13 and its derivatives have the potential as alternative agents against *H. pylori* infection and alleviate inflammatory response.

**Supplementary Information:**

The online version contains supplementary material available at 10.1186/s12866-022-02701-z.

## Background

Lactic acid bacteria (LAB) are a group of gram-positive cocci or rods, which produce lactic acid as the major end product of the fermentation of carbohydrates. *Lactobacillus*, *Lactococcus*, *Leuconostoc*,*Pediococcus*, *Streptococcus*, and *Enterococcus*, are the main genera of LAB with probiotic activity [1]. The biological activity of LAB, its derivative exopolysaccharide (EPS), and bacteriocins have been shown to prevent infections, against tumor progression, modulate the immune response, relieve allergies, alter the microbiome, and alleviate lactose intolerance [2–6].

We previously isolated a high EPS-producing *Lactiplantibacillus* *pentosus* strain, SLC13, from mustard pickles in Taiwan [4, 7]. SLC13 showed high inhibitory activity against the growth of *Staphylococcus aureus*, *Enterococcus faecalis*, and *Yersinia enterocolitica* [4]. The complete genome sequence of SLC13 revealed a plantaricin gene cluster, which is responsible for bacteriocins biosynthesis, and could be associated with its broad-spectrum antimicrobial activity [7].

*Helicobacter pylori* is a gram-negative and microaerophilic bacterium and infects approximately 50% of the world's population [8]. Importantly, persistent *H. pylori* infection is associated with the development of gastrointestinal diseases, including gastric adenocarcinoma [8]. *H. pylori* urease activity, flagella-mediated motility, and adhesins, are required for the neutralization of hostile acidic conditions and successful colonization [9]. Triple therapies consisting of a proton pump inhibitor (PPI), clarithromycin, metronidazole, levofloxacin, or amoxicillin, are the most common treatments for *H. pylori* infections [10]. However, the increase in antibiotic resistance of *H. pylori* has been reported to reduce the cure rates of *H. pylori* therapies [10, 11]. Therefore, In this study, we aimed to evaluate the anti-*H. pylori* activity of *L. pentosus* SLC13 to determine potential applications of SLC13 in the future.

## Results

### Growth of lactobacilli under simulated gastrointestinal conditions

To test and compare the growth rate of lactobacilli strains under simulated gastrointestinal conditions, lactobacilli were cultured in MRS broth, acidic MRS broth (pH 3.0), and 0.3% bile salt in MRS broth. Our results showed that all three strains were tolerant to gastrointestinal challenges (Fig. S1B & S1C). However, SLC13 grew faster than LGG and BCRC 14619 T in MRS broth and acidic MRS broth (Fig. S1A & S1B). In addition, to test the ability of three lactobacilli strains to withstand the extremely acidic conditions of the gastric cavity, the survival of lactobacilli was determined by counting the viable cells after three hours incubation in MRS broth at pH 2. The results showed that only SLC13 had a high survival rate (90.4%) under extremely acidic conditions (Fig. S2).

### Adhesion ability and cytotoxicity of lactobacilli to AGS and GES-1 cells

**Fig. 1:**
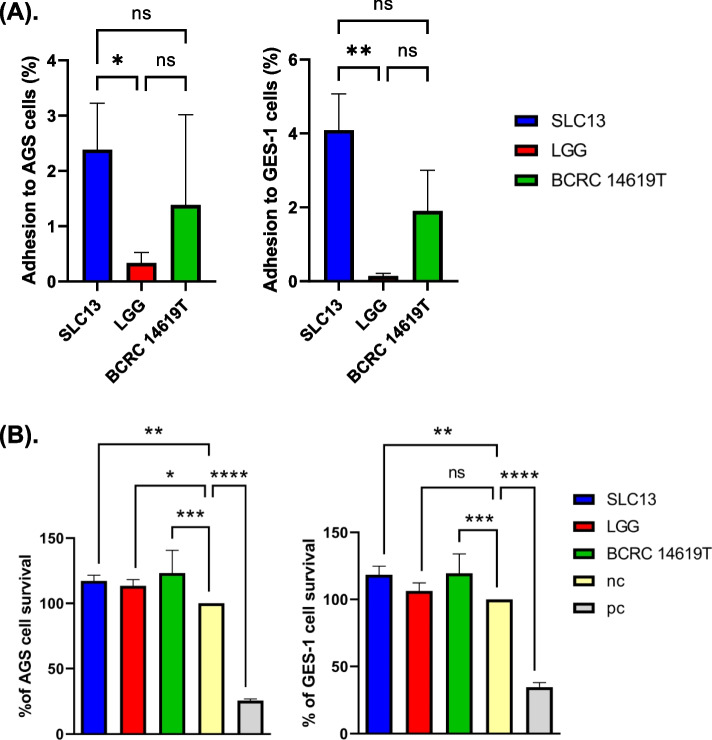
Adhesion and cytotoxicity of SLC13, LGG, and BCRC 14619 T to AGS and GES-1. **A** SLC13, LGG, and BCRC 14619 T adhesion to AGS and GES-1 cells. **B** Cell viability was determined by MTT assay after 4 h of coculture (MOI = 100). Error bars represent the standard deviation of biological triplicates. *, *p* < 0.05; **, *p* < 0.01; ***, *p* < 0.001; ****, *p* < 0.0001; ns, no significant difference; pc, positive control (Triton X-100 treatment); nc, negative control (AGS or GES-1 cells only, without treatment)

The adhesion rates of SLC13, LGG, and BCRC 14619 T to AGS and GES-1 cells altered depending on the tested strains which ranged from 0.33 to 2.38% (AGS cells) and from 0.14 to 4.09% (GES-1 cells), respectively (Fig. 1A). Among the three strains examined, SLC13 showed the highest adhesion ability as compared to LGG and BCRC 14619 T (Fig. 1A). Consistent with the colony counting results, we observed that more SLC13 adhered to AGS and GES-1 cells, compared to LGG and BCRC 14619 T under the microscope (Fig. S3).

To test the safety of *Lactobacillus* in future applications, we performed the cell viability assay to determine the cytotoxicity of three lactobacilli strains to AGS and GES-1 cells (Fig. 1B). Cells treated with 0.1% Triton X-100 inhibited the proliferation of these cells significantly and thus served as a positive control in this assay (Fig. 1B). The results showed that the coculture of SLC13, LGG, and BCRC 14619 T with cells for 4 h had no significant cytotoxic effects on cells (Fig. 1B).

### Antibacterial activity of lactobacilli against *H. pylori*

**Fig. 2:**
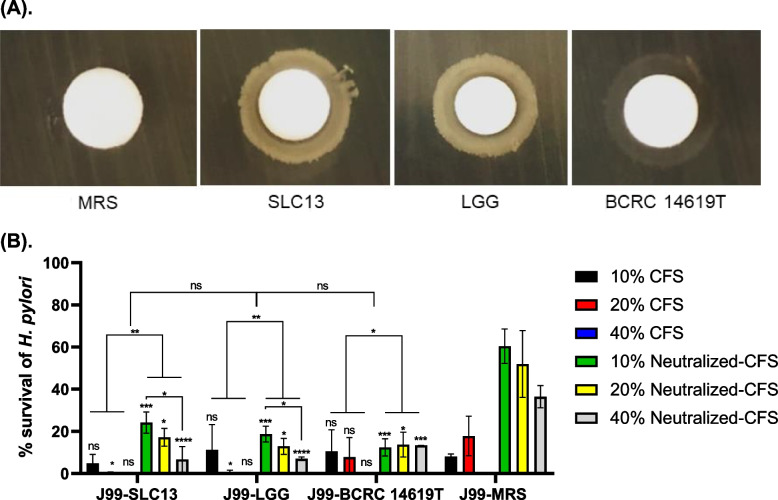
Anti-*H. pylori* activity of lactobacilli. **A** The inhibitory effect of lactobacilli on *H. pylori* J99 was determined by disk diffusion method. MRS broth alone was used as a negative control. **B** The survival rate of *H. pylori* in the presence of different concentrations (10%, 20%, and 40%) of CFS or neutralized-CFS (adjusted to pH 6.5 by NaOH) at microaerophilic conditions for 4 h. The viability of *H. pylori* after 72 h co-incubation with CFS was evaluated by determining the viable bacterial count on Brucella agar containing 10% horse serum plates after incubation at 37 °C under microaerophilic conditions. MRS broth pH 4.0 (acid-CFS control) and pH 6.5 (neutralized-CFS control) were used as controls of the reaction. CFS, cell-free supernatant. Error bars represent the standard deviation of biological triplicates. *, *p* < 0.05; **, *p* < 0.01; ns, no significant difference

Our previous study showed that SLC13 had antibacterial activity against the growth of *S. aureus*, *E. faecalis*, and *Y. enterocolitica* [4]. Therefore, we performed the disk diffusion method to evaluate and compare the inhibitory activity of SLC13, LGG, and BCRC 14619 T against *H. pylori* reference strain J99 (Fig. 2A). Our results indicated that all tested strains exhibited an inhibitory effect against *H. pylori* J99, as demonstrated by forming the zones of growth inhibition (Fig. 2A).

To test the anti-*H. pylori* activity of cell-free supernatant (CFS) collected from lactobacilli strains tested and neutralized-CFS (CFS with pH neutralization), we recorded the pH value of collected CSF from three lactobacilli strains to prevent the different acidities of CSF from affecting the phenol red indicator to determine *H. pylori* urease activity. The mean pH value of SLC13, LGG, and BCRC 14169 T CSF was 3.96, 3.96, and 4.12, respectively. In addition, we added ~ 80 μL NaOH (5 M) to neutralize the pH value of 10 mL collected acidic CSF to pH 6.5 to prevent the dilution effect. The survival rate of *H. pylori* J99 in the presence of CFS of lactobacilli was further determined, and the results showed that the CFS and neutralized-CFS (CFS with pH neutralization) of lactobacilli with various concentrations (10%, 20%, and 40%) showed different inhibition activity to *H. pylori* growth (Fig. 2B). In general, nearly all CFS showed higher anti-*H. pylori* activity compared to the neutralized-CFS (except 10 and 20% CFS of BCRC 14619 T) (Fig. 2B). Only 22%, 19%, and 13% of *H. pylori* survived under the treatment with 10% neutralized-CFS of SLC13, LGG, BCRC 14619 T, respectively (Fig. 2B). Moreover, the antibacterial activity of CFS from three lactobacilli strains was not significantly different (*p* > 0.05).

### Effect of lactobacilli CFS on urease activity of *H. pylori*

**Fig. 3:**
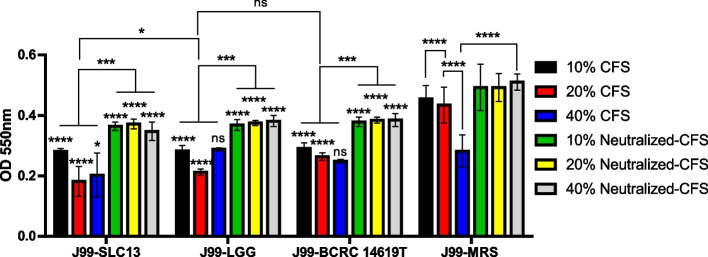
Inhibition of urease activity of lactobacilli cell-free supernatant. Effect of lactobacilli CFS and neutralized-CFS (10%, 20%, and 40% concentrations) on urease activity of *H. pylori* was determined by measuring the absorbance of ammonium at 550 nm after 4 h co-incubaction. MRS broth pH 4.0 (acid-CFS control) and pH 6.5 (neutralized-CFS control) were used as controls of the reaction. CFS, cell-free supernatant. Error bars represent the standard deviation of biological triplicates (with technical replicates). *, *p* < 0.05; ***, *p* < 0.001; ****, *p* < 0.0001; ns, no significant difference

Nearly all CFS showed higher antibacterial activity compared to neutralized-CFS (Fig. 2B). The expression level and activity of urease are critical for *H. pylori* survival under an acidic environment. Therefore, we further evaluated the effects of lactobacilli CFS on the urease activity of *H. pylori* (Fig. 3). Overall, the results showed that the urease activity of *H. pylori* was decreased by treating *H. pylori* with CFS and neutralized-CFS collected from three examined strains (Fig. 3). The anti-urease activity of *H. pylori* showed a dose-dependence of the lactobacilli CFS concentrations. Among three tested strains, the CFS of SLC13 showed the highest urease inhibitory activity, compared to the CFS of LGG and BCRC 14619 T. In addition, neutralized-CFS had a less potential ability to decrease urease activity compared to CFS (Fig. 3).

### Anti-*H. pylori* adhesion activity of lactobacilli

**Fig. 4:**
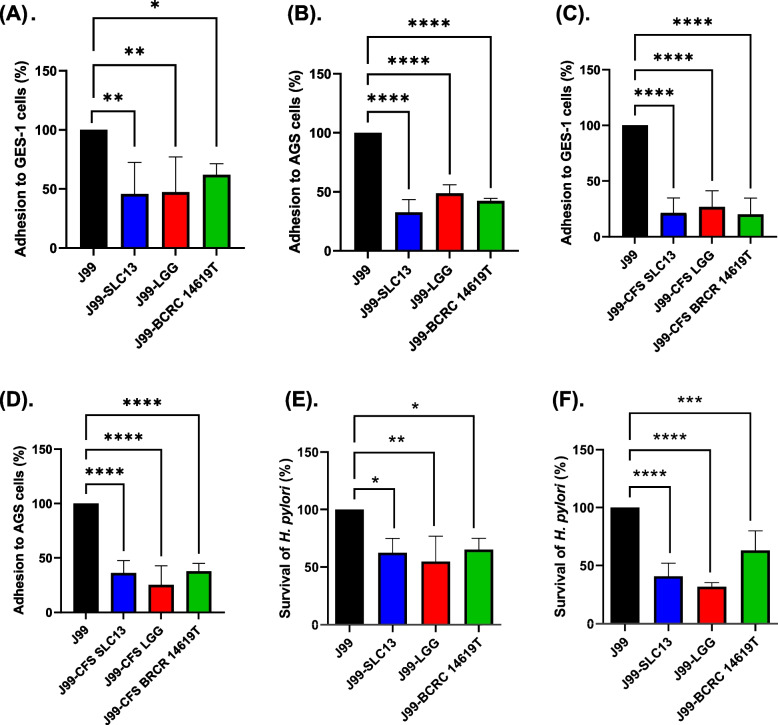
Anti-*H. pylori* adhesion activity of lactobacilli. Adhesion of *H. pylori* to GES-1 (**A**) and AGS cells (**B**) after pretreatment with lactobacilli for 30 min. Adhesion of *H. pylori* to GES-1 (**C**) and AGS cells (**D**) in the presence of lactobacilli CFS simultaneously. Survival of *H. pylori* in the presence of lactobacilli CFS simultaneously in RPMI cell culture medium for GES-1 cells (**E**) and F12 culture medium for AGS cells (**F**). *H. pylori* J99 alone was used as a control in these assays. Error bars represent the standard deviation of biological triplicates. *, *p* < 0.05; **, *p* < 0.01; ***, *p* < 0.001; ****, *p* < 0.0001; ns, no significant difference

The adhesion ability of *H. pylori* to the surface of gastric epithelial cells is an important initial step towards successful colonization [9]. Therefore, we investigated whether lactobacilli or their derived CFS suppress the adhesion of *H. pylori* to AGS and GES-1 cells (Fig. 4A-D). The results showed that pretreatment with SLC13, LGG, and BCRC 14619 T for two hours exhibited a reduction of *H. pylori* adhesion to GES-1 cells by 54.2%, 52.4%, and 38.0%, respectively (Fig. 4A). Moreover, the adhesion of *H. pylori* to AGS cells was reduced by the pretreatment with SLC13 (67.3%), LGG (51.2%), and BCRC 14619 T (57.7%) (Fig. 4B).

We further determined whether lactobacilli CFS has the anti-adhesion activity of *H. pylori* to GES-1 and AGS cells (Fig. 4C & D). The results showed that the adherence of *H. pylori* to GES-1 and AGS cells was dramatically reduced in the presence of lactobacilli CFS (Fig. 4C & D). The coculture of cells with CFS of SLC13, LGG, and BCRC 14619 T simultaneously caused a reduction of *H. pylori* adhesion to GES-1 cells by 78.7%, 73.2%, and 79.9%, respectively (Fig. 4C). Moreover, the adhesion of *H. pylori* to AGS cells was reduced by coculture with the CFS of SLC13 (63.7%), LGG (74.6%), and BCRC 14619 T (62.0%) (Fig. 4D).

To assess whether the inhibitory effect of lactobacilli CFS on adherence of *H. pylori* to GES-1 and AGS is associated with the reduction of *H. pylori* amount, we evaluated the survival rate of *H. pylori* with lactobacilli CFS treatment. Our results showed that the CFS of SLC13, LGG, and BCRC 14619 T strains killed *H. pylori* and resulted in a reduction of *H. pylori* adhesion to host gastric epithelium cells (Fig. 4E & F).

### Effect of lactobacilli on *H. pylori*-induced Inflammation

**Fig. 5:**
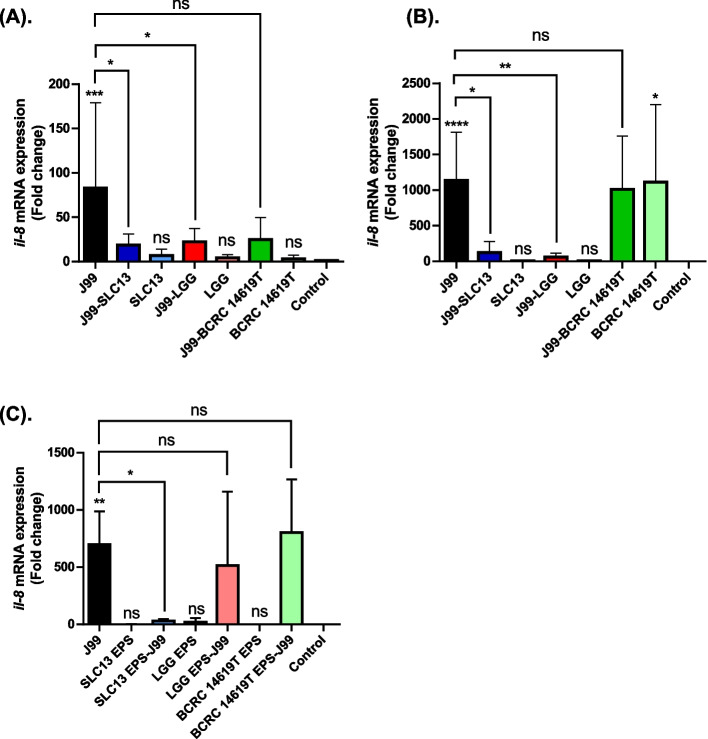
Anti-*H. pylori*-induced inflammation of lactobacilli and their derived exopolysaccharide. GES-1 (**A**) and AGS (**B**) cells were pretreated with lactobacilli SLC13, LGG, or BCRC 14619 T for 2 h, and then cells were infected with *H. pylori* J99 (MOI = 100) for 2 h. **C**. AGS cells were treated with EPS extracted from lactobacilli SLC13, LGG, or BCRC 14619 T at a concentration of 500 ng/mL with *H. pylori* J99 (MOI = 100) simultaneously for 2 h. After *H. pylori* infection, RNA was extracted from cells to determine the *il-8* gene expression. AGS and GES-1 cells alone without any treatments were used as a negative control to normalize the measurements. Error bars represent the standard deviation of biological triplicates. *, *p* < 0.05; **, *p* < 0.01; ***, *p* < 0.001; ****, *p* < 0.0001; ns, no significant difference; EPS, exopolysaccharide

*H. pylori* infection induces IL-8 production and contributes to inflammation of gastric epithelial cells [12]. We therefore determined whether the lactobacilli and their derived EPS can inhibit *H. pylori*-induced IL-8 gene expression of AGS and GES-1 cells by using RT-qPCR (Fig. 5A & B). The treatment of GES-1 and AGS cells with lactobacilli SLC13, LGG, and BCRC 14619 T, did not induce IL-8 mRNA expression in cells, compared to cells infected with *H. pylori* (Fig. 5A & B). Moreover, the pretreatment of three lactobacilli strains effectively reduced the IL-8 mRNA in GES-1 infected with *H. pylori* (Fig. 5A). In AGS cells, pretreatment with SLC13 and LGG effectively reduced the IL-8 mRNA which was induced by *H. pylori* infection (Fig. 5B). Interestingly, BCRC 14,619 exhibited a difference of IL-8 induction in GES-1 and AGS cells (Fig. 5A & B). Significant induction of IL-8 mRNA was detected in AGS cells treated with BCRC 14,619 (Fig. 5B).

Lactobacilli-derived EPS have been shown to downregulate the inflammatory cytokine expression [13]. Therefore, we next determined the EPS production in three tested lactobacilli strains, and the results showed that all three strains produced high EPS (Fig. S4). Compared to AGS cells, GES-1 infected with *H. pylori* induced lower IL-8 expression, therefore, we only tested the immune suppression activity of EPS extracted from three lactobacilli strains in AGS cells infected with *H. pylori* (Fig. 5C). The results showed that EPS derived from three strains did not induce IL-8 expression in AGS (Fig. 5C) and did not have anti-*H. pylori* activity (Fig. S5). Moreover, only treatment of AGS cells with SLC13 EPS significantly decreased IL-8 expression induced by *H. pylori* infection as compared to cells treated with LGG and BCRC 14619 T EPS (Fig. 5C).

## Discussion

The increasing prevalence of antibiotic resistance in *H. pylori* leads to a decrease in eradication rate with standard triple therapy, including PPI, amoxicillin, and clarithromycin [14]. Moreover, antibiotic treatments have been shown to cause gut microbiota dysbiosis and subsequently alter immune cell function, which is associated with reduced synaptic transmission and gamma oscillations in the hippocampus [15]. Therefore, probiotics are considered as an alternative or adjuvant treatment to improve *H. pylori* eradication and reduce drug side effects [16, 17]. In this study, we characterized SLC13 and demonstrated it had the potential to against *H. pylori* infection and *H. pylori*-induced inflammation.

Although SLC13 was isolated from fermented food, our results showed that SLC13 had a high tolerance to gastrointestinal challenge (Fig. S1), colonized greatly to the gastric epithelial AGS and GES-1 cells (Fig. 1A & B), and did not cause cytotoxicity to cells (Fig. 1C). Importantly, SLC13 and its derived CFS displayed a strong anti-*H. pylori* J99 activity (Fig. 2). However, it remains to study whether SLC13 had broad-spectrum activity against multidrug-resistant *H. pylori*. Zheng et al. reported that lactobacilli LPS16 had anti-*H. pylori* activity depended on the secreted components and lactic acid-mediated bactericidal activity [18]. Our results showed that compared to neutralized CFS, acidic CFS had higher anti-*H. pylori* activity (Fig. 2B). These results suggest that SLC13 against *H. pylori* by acidifying culture medium in part; however, the other secreted components in the CFS also showed the antibacterial activity against *H. pylori.* Lactic acid causes membrane permeabilization in gram-negative bacteria, which in addition to its antimicrobial property, can promote the bactericidal effects of other compounds such as bacteriocins, which have been shown to have strong antibacterial activity against gram-negative pathogens, including *H. pylori* [19, 20]. The complete genome sequence of SLC13 revealed a plantaricin gene cluster, which may contribute to bacteriocins biosynthesis [7]. However, the characteristics and regulation of bacteriocins of SLC13 remain to be studied.

Urease activity is an important virulence factor for *H. pylori* colonization, which changes the viscoelastic nature of mucus by neutralizing acidic pH [21]. Our results revealed that CFS reduced the urease activity of *H. pylori* J99. In addition, the urease inhibitory effect of CFS was more efficient as compared to the neutralized-CFS (Fig. 3). We found that *H. pylori* treated with CFS had a lower survival rate, compared to the neutralized-CFS (Fig. 2). These results suggest that the decrease in urease activity is caused by the reduction of live bacteria. However, the treatment of *H. pylori* with neutralized-CFS showed a lower urease activity, compared to the control group (Fig. 3). Ryan et al. reported that the expression of 8 of 12 Cag pathogenicity island genes was downregulated by exposure to *Ligilactobacillus salivarius* and resulted in the loss of functionality of the Cag secretion system [22]. *L. rhamnosus* JB3 showed the ability to suppress the association of *H. pylori* to cells and its induced IL-8 levels, as well as the mRNA expression of *vacA*, *sabA*, and *fucT* of *H. pylori* [23]. Therefore, it is worth studying whether the SLC13 and its derived CFS modulate the gene expression in *H. pylori* and thus reduce the adhesion ability and urease activity of *H. pylori*.

The anti-*H. pylori* adhesion to cells of lactobacilli can be achieved by competition for binding sites on epithelial cells, release antimicrobial compounds to kill *H. pylori*, and down-regulation of adhesin gene expression. Previous results showed that lactobacilli acted directly on *H. pylori* by an effector molecule released into the medium and inhibited the expression of the adhesion-encoding gene *sabA* [24]. Our results showed that both live SLC13 and its CFS suppressed the adherence of *H. pylori* to GES-1 and AGS cells (Fig. 4). Furthermore, the Giemsa staining displayed that SLC13 did not adhere efficiently to cells as compared to *H. pylori* (data not shown). These results suggest that the bacteriocin or unknown compounds secreted by SLC13 majorly contribute to the reduction of *H. pylori* adherence to GES-1 and AGS cells. Jin et al. reported that a combination of *L. salivarius* LN12 CFS with amoxicillin and clarithromycin destroyed the *H. pylori* biofilm to a greater extent than when separately [25]. Therefore, the effects of SLC13 in combination with other antibiotics on *H. pylori* motility, biofilm, and toxin production, are worth investigating.

*H. pylori* can directly translocate CagA into host cells, which leads to the production of IL-8 by the epithelial cells [26]. In addition, nucleotide-binding oligomerization domain 1 (NOD1) recognizes *H. pylori*-derived peptidoglycan or outer membrane vesicles intracellular that trigger the pro-inflammatory signaling cascade through nuclear translocation of NF-κB subunits in gastric epithelial cells [27]. In this study, we demonstrated that SLC13 contained potential activity to inhibit *H. pylori* adhesion to gastric epithelial cells, which in turn, may attenuate CagA translocation and IL-8 production in cells (Fig. 5A & B). EPS have been reported to reduce TNF-α, IL-8, and MCP-1 in the gastric mucosa of *H. pylori*-infected mice [28]. We showed that SLC13, LGG, and BCRC 14619 T are high EPS-producing strains, however, compared to the SLC13 EPS, the EPS extracted from LGG and BCRC 14619 T did not reduce the IL-8 expression in AGS cells infected with *H. pylori* (Fig. 5C). The difference in biological properties of EPS extracted from three tested strains suggests that the structure and composition of their EPS are strain-specific [29]. Therefore, the mechanisms through which SLC13 and its derivatives modulate the pro-inflammatory signaling are worth investigating.

In previous clinical trial results, Chen et al. reported that the probiotics containing *Lactobacillus acidophilus* and *L. rhamnosus* can reduce the *H*. *pylori* bacterial load, but *H*. *pylori* can not be eradicated by probiotics alone [30]. However, these two lactobacilli strains used in their study are commercially available probiotics by the Taiwan Sugar Corporation. In contrast, SLC13 and previously reported JB3 with anti-*H. pylori* activity were isolated from fermented food and dairy food, respectively [4, 31]. At present, these two strains are not commercially available probiotics. Although we showed the in vitro anti-*H. pylori* activity of SLC13, an in-depth study is required to determine the characteristics (including the safety in use) of SLC13 before examining its functionality in animal models and human clinical trials. *H. pylori* infections were related to several extragastric diseases such as cardiovascular, hematological, and neurological conditions [32]. Probiotics generate bacteriostatic chemicals that limit *H. pylori* colonization and minimize treatment-related adverse effects such as antibiotic-associated diarrhea [33]. Therefore, the effects of SLC13 on *H. pylori*-related extragratric diseases with reduced antibiotic-causing adverse effects should be studied.

## Conclusions

Our results showed that *L. pentosus* SLC13 had no cytotoxicity, high tolerance to acid and bile salt, and high cell adhesion ability. Moreover*,* SLC13 and its derivatives in CFS and EPS displayed multiactivity against colonization and pathogenesis of *H. pylori* through modulating inflammatory response, reducing urease activity and attachment on the cells, and killing *H. pylori*. Therefore, SLC13 has the potential as a preventive agent or adjuvant treatment for *H. pylori* infection. However, it is necessary to verify the safety and anti-*H. pylori* activity of SLC13 by animal models in the future.

## Material and methods

### Bacterial strains and culture conditions

*Lactiplantibacillus* *pentosus* SLC13, *Lactobacillus gasseri* BCRC 14619 T, and *Lacticaseibacillus rhamnosus* LGG were stored at − 80 °C in De Man, Rogosa and Sharpe (MRS) broth containing 16% glycerol (v/v) until tested. LGG and BCRC 14619 T are widely used probiotic strains and are thus used as control strains in this study [34, 35]. LGG and BCRC 14619 T were gifts from Dr. Ying-Chieh Tsai at National Yang Ming Chiao Tung University (Taiwan). SLC13 was the laboratory stock strain isolated from mustard pickles in Taiwan [4]. To activate lactobacilli, the bacteria were cultured in MRS broth at 37 °C for 24 h and then subjected to each experimental study. *H. pylori* J99 was stored at − 80 °C in Brucella broth containing 20% glycerol (v/v) until tested. *H. pylori* was grown on Brucella agar plates containing 10% (v/v) horse serum (Gibco BRL, Life Technologies, Rockville, MD) at 37 °C in microaerophilic conditions (5% O_2_, 10% CO_2_, and 85% N_2_).

### Cell culture

AGS cells (human gastric adenocarcinoma epithelial cells, ATCC CRL 1739) and GES-1 cells (normal human gastric epithelial cells) were gifts from Dr. Hsiu-Chi Cheng at National Cheng-Kung University Hospital (Taiwan). AGS and GES-1 cells were maintained in Ham′s F12 and RPMI 1640 medium supplemented with 10% fetal bovine serum (FBS) (Gibco BRL, Life Technologies, Rockville, MD), penicillin (100 IU/mL), and streptomycin (100 µm/mL) at 37 °C with 5% CO_2_, respectively.

### Cell adhesion assay

AGS and GES-1 cells (3 × 10^5^ cells) were seeded into 12-well plates in F12 and RPMI-1640 media, respectively, with 10% FBS and 1% penicillin–streptomycin, and grown to a monolayer at 37 °C for 24 h. Before performing infection, the culture medium was replaced with fresh medium without antibiotics. To determine the adhesion ability of lactobacilli, cells were infected with a multiplicity of infection (MOI) of 100 of lactobacilli and incubated at 37 °C for 90 min. For lactobacilli-*H. pylori* co-culture, the cells were incubated with lactobacilli strains (MOI = 100) 30 min before being infected with *H. pylori* (MOI = 100) and then incubated at 37 °C for 1 h. For lactobacilli CFS-*H. pylori* co-culture, lactobacilli were cultured in cell culture medium for 4 h. After centrifugation at 3,011 xg for 10 min, the supernatant was collected and filtered by a 0.22 µm diameter filter. Sodium hydroxide (NaOH) was used as the neutralizing agent to adjust the pH of CFS to 6.5 (neutralized-CFS). The cells were then incubated with *H. pylori* (MOI = 100) and collected CFS at different concentrations simultaneously at 37 °C for 90 min. The cells were washed three times with PBS, lysed by incubation with 0.1% saponin at 37 °C for 15 min, and plated on MRS plates or Brucella plates with 10% horse serum. The colonies were counted to determine the number of adherent bacteria. The percentage of bacterial adhesion to cells was calculated according to the following formula: adhesion ability (%) = (adherent bacteria)/(total bacteria) × 100. Cell adhesion assay was conducted in biological triplicate to ensure reproducibility.

### Cytotoxicity on AGS and GES-1 cells

The cytotoxicity effect of lactobacilli on AGS and GES-1 cells was determined by 3-(4,5-Dimethylthiazol-2-yl)-2,5-diphenyltetrazolium bromide (MTT; Sigma-Aldrich, St Louis, MO, USA) assay according to the previous study [36]. Cells (1.5 × 10^5^) were grown and allowed to adhere to a 96-well plate at 37 °C for 24 h to approximately 80% confluence and then cells were treated with lactobacilli with a MOI of 100 for 4 h. The medium was removed and the cells were washed three times with PBS. Thirty µL of MTT reagent (5 mg/mL) was added to each well and the plates were incubated at 37 °C for 3 h in the dark. Two hundred μL of DMSO was added to each well and the plate was incubated with shaking (150 rpm) at room temperature for 15 min to allow the color to develop. The OD was measured at 570 nm. Cell viability (%) = (A_sample_/A_control_) × 100, where A_sample_ is the absorbance of the cells that were incubated with the medium containing lactobacilli or 0.1% Triton X-100, and A_control_ is the absorbance of the cells alone. The cytotoxicity assay was conducted in biological triplicate to ensure reproducibility.

### Anti-*H. pylori* activity of lactobacilli

Anti-*H. pylori* activity of lactobacilli was determined by the disk diffusion method as previously described [37]. Briefly, *H. pylori* suspension (OD_600_ = 2 in Brucella broth) was spread on Brucella agar plates containing 10% horse serum. The overnight culture of lactobacilli was adjusted to OD_600_ = 1 and added to paper disks (Toyo Roshi Kaisha, Ltd, Japan) with a diameter of 6 mm. The plates were cultured under microaerophilic conditions for 72 h and the inhibition zone was determined in diameter.

For co-culture assay, *H. pylori* suspensions were adjusted to OD_600_ = 0.2 in MRS broth containing 10% horse serum and were incubated under microaerophilic conditions at 37 °C with 10%, 20%, or 40% lactobacilli CFS or neutralized-CFS for 4 h. MRS broth was used as the control. The viability of *H. pylori* after 4 h co-incubation with CFS was evaluated by determining the viable bacterial count on Brucella agar containing 10% horse serum plates after incubation at 37 °C under microaerophilic conditions. MRS broth (pH 4.0 and pH 6.5) was used as a control of the reaction. *H. pylori* survival rate (%) = [A1(Log CFU/mL)/A0(Log CFU/mL)] × 100, where A0 is the viable count of *H. pylori* at 0 h and A1 is the viable count of *H. pylori* after 4 h co-incubation with CFS. Anti-*H. pylori* activity of lactobacilli was conducted in biological triplicate to ensure reproducibility.

### Urease activity assay

To analyze the inhibitory effects of *H. pylori* urease by CFS of lactobacilli, *H. pylori* cells were resuspended in Brucella broth (OD_600_ = 0.2) and mixed with 10, 20, or 40% lactobacilli CFS or neutralized-CFS, and then incubated at 37 °C for 4 h. Urease activity was determined in phosphate buffer containing urea buffer (25 mM urea and 0.012% phenol red in PBS) according to the previous study [38–40]. The resulting color was read at OD_550_ after 2 h incubation. MRS broth (pH 4.0 and pH 6.5) was used as a control of the reaction. The urease activity assay was conducted in biological triplicate along with technical triplicate to ensure reproducibility.

### Exopolysaccharide extraction

Lactobacilli were inoculated in 25 mL MRS broth containing 2% sucrose at 37 °C for 24 h. Bacterial cultures were centrifuged at 3,011 xg for 30 min to remove cells and their debris. EPS were precipitated from supernatants by adding 4 volumes of 95% ethanol, and the mixture was incubated at 4 °C for 24 h [41]. After ethanol precipitation, the samples were centrifugated at 3,011 xg for 30 min, and the supernatant was removed. The precipitate of pure EPS was dried in the oven at 60 °C for 24 h. The precipitates were re-suspended in distilled water, filtered by 0.45 µm diameter filter, and stored at -80 °C until testing. The production of EPS was analyzed by the phenol–sulfuric method using glucose as a reference standard [42]. EPS production assay was conducted in biological triplicate to ensure reproducibility.

### IL-8 mRNA determination by reverse-transcription quantitative PCR (RT-qPCR)

To determine the anti-inflammation ability of lactobacilli, AGS and GES-1 cells were pretreated with lactobacilli (MOI = 100) for 2 h, and then cells were infected with *H. pylori* (MOI = 100, 2 h). In contrast, to determine the anti-inflammation activity of EPS derived from lactobacilli, AGS cells were treated with lactobacilli EPS (500 ng/mL) and *H. pylori* (MOI = 100) simultaneously for 2 h. After *H. pylori* infection for 2 h, Trizol reagent was used for total mRNA extraction. A total of 1 μg RNA was reverse-transcribed with SuperScript II RNase H-reverse transcriptase (Bionovas biotechnology Co., Ltd, Canada) for cDNA synthesis. The amounts of IL-8 mRNA were analyzed by RT-qPCR performed in a StepOnePlus™ system (Themo Fisher Scientific Inc, USA) with the SYBR Green Master Mix (Ampliqon, Denmark) in qPCR 96 well plates (MB-Q96-LP and MB-QSM; Gunster Biotech, New Taipei City, Taiwan). The primers IL-8-forward (5′-ACTGAGAGTGATTGAGAGTGGAC-3′) and IL-8-reverse (5′- AACCCTCTGCACCCAGTTTTC − 3′) and β-actin-forward (5’-GACCTCTATGCCAACACAGT-3’) and β-actin-reverse (5'-AGTACTTGCGCTCAGGAGGA-3') were used for IL-8 and β-actin detection, respectively [43, 44]. Samples were normalized to the level of β-actin mRNA. The RT-qPCR was performed in triplicate in a total reaction volume of 25 μL containing 12.5 μL of SYBR Green PCR Master Mix, 0.5 μL of forward and reverse primers, 10.5 μL of distilled H_2_O, and 1 μL of cDNA from each sample. Samples were heated for 15 min at 95 °C and amplified for 40 cycles of 15 s at 95 °C and 60 s at 60 °C. Quantification was performed using the 2-ΔΔCt method [45], where the Ct value was defined as the threshold cycle of PCR at which amplified product was detected. The ΔCt was obtained by subtracting the housekeeping gene (β-actin) Ct value from the Ct value of the IL-8 gene. The fold change was calculated according to the formula 2-ΔΔCt, where ΔΔCt was the difference between ΔCt and the ΔCt calibrator value (which was assigned a value of 1 arbitrary unit). IL-8 mRNA expression level was conducted by RT-qPCR in biological triplicate to ensure reproducibility.

### Statistical analysis

The data were expressed as a mean of three replicates ± SD. ANOVA one-way test was used to calculate the statistical significance of the experimental results between two groups. In the urease activity assay and anti-*H. pylori* activity of lactobacilli assay, ANOVA two-way test was used to assess multiple comparisons in those groups. A *p*-value less than 0.05 was considered as a significant difference.

## Supplementary Information

**Additional file 1: Figure S1.** Growth curves of SLC13, LGG, and BCRC 14619T incubated for 24 h in MRS broth (pH 6.5) (A), acidic MRS broth (pH 3.0) (B), and MRS broth with 3% bile salts (pH 6.5) (C). Error bars represent the standard deviation of biological triplicates. **Figure S2.** Survival of SLC13, LGG, and BCRC 14619T was determined by counting the viable cells after 3 hours incubation in MRS broth (pH 2.0). Error bars represent the standard deviation of biological triplicates. The survival rate of LGG and BCRC 14169T in acid MRS after 3 hours incubation was compared to SLC13 in acid MRS after 3 hours incubation. ****, *p *< 0.0001. **Figure S3.** Adhesion of SLC13, LGG, and BCRC 14619T to AGS and GES-1 cells. The microscope observation of attachment of lactobacilli to AGS and GES-1 cells. The bacteria were stained with Giemsa and thus showed purple color. **Figure S4.** Extraction of lactobacilli exopolysaccharide. Exopolysaccharide production in the culture medium of lactobacilli SLC13, LGG, and BCRC 14619T in MRS broth containing 2% sucrose at 37^o^C for 24 h. Phenol-sulfuric acid method was used to measure the content of EPS using glucose as standard. EPS content was calculated according to the regression equation based on the standard curve, and then converted with dilution ratio. EPS, exopolysaccharide. NC, negative control (detection background of phenol-sulfuric mixture). Error bars represent the standard deviation of biological triplicates. ****, *p* <0.0001. **Figure S5.** Anti-H. pylori activity of lactobacilli exopolysaccharide.

## Data Availability

The datasets used and/or analysed during the current study available from the corresponding author on reasonable request.
