# Supplementary material for: Anti-Helicobacter pylori activity of potential probiotic Lactiplantibacillus pentosus SLC13
Source: BMC Microbiol. 2022 Nov 21;22:277. doi: 10.1186/s12866-022-02701-z (PMC9677914; doi:10.1186/s12866-022-02701-z)
Supplement: Supplementary file 1 — Additional file 1: Figure S1. Growth curves of SLC13, LGG, and BCRC 14619T incubated for 24 h in MRS broth (pH 6.5) (A), acidic MRS broth (pH 3.0) (B), and MRS broth with 3% bile salts (pH 6.5) (C). Error bars represent the standard deviation of biological triplicates. Figure S2. Survival of SLC13, LGG, and BCRC 14619T was determined by counting the viable cells after 3 hours incubation in MRS broth (pH 2.0). Error bars represent the standard deviation of biological triplicates. The survival rate of LGG and BCRC 14169T in acid MRS after 3 hours incubation was compared to SLC13 in acid MRS after 3 hours incubation. ****, p < 0.0001. Figure S3. Adhesion of SLC13, LGG, and BCRC 14619T to AGS and GES-1 cells. The microscope observation of attachment of lactobacilli to AGS and GES-1 cells. The bacteria were stained with Giemsa and thus showed purple color. Figure S4. Extraction of lactobacilli exopolysaccharide. Exopolysaccharide production in the culture medium of lactobacilli SLC13, LGG, and BCRC 14619T in MRS broth containing 2% sucrose at 37oC for 24 h. Phenol-sulfuric acid method was used to measure the content of EPS using glucose as standard. EPS content was calculated according to the regression equation based on the standard curve, and then converted with dilution ratio. EPS, exopolysaccharide. NC, negative control (detection background of phenol-sulfuric mixture). Error bars represent the standard deviation of biological triplicates. ****, p <0.0001. Figure S5. Anti-H. pylori activity of lactobacilli exopolysaccharide. [file 12866_2022_2701_MOESM1_ESM.doc]

**Methods**

**Acid and bile salt tolerance**

The growth rate of lactobacilli under simulated gastrointestinal challenges was determined according to the previous study with modification [1]. The growth rate of bacteria was determined over 24 h at 37°C using a BioTek Synergy HTX multimode reader. A total of 200 μL of sterile MRS (pH 3 or 6.5) or MRS supplemented with 0.3% bile salts, was dispensed into sterile 96-well microplates and inoculated with an overnight culture of each strain to give a ﬁnal inoculum of 1%. Readings were taken every 15 min of absorbance of each well (scanned at OD_600nm_) in the microplates over the 24 h period. To test the survival of lactobacilli in an extremely acid environment (pH 2), the bacteria were incubated at 37°C and the viable organisms were counted after exposure to acid conditions for 3 h on MRS agar incubated at 37^o^C for 48 hours. The survival cell count was calculated according to the number of colonies grown on MRS agar, compared to the initial bacterial concentration. Lactobacilli survival rate (%) = [A1(Log CFU/mL)/A0(Log CFU/mL)] × 100, where A0 is the viable count of lactobacilli at 0 h and A1 is the viable count of lactobacilli after 3 h incubation in acidic MRS broth (pH 2). Each strain was analyzed in triplicate wells on at least three separate occasions.

**Cell adhesion assay**

AGS and GES-1 cells (3x10^5^ cells) were seeded into 12-well plates in F12 and RPMI-1640 media, respectively, with 10% FBS and 1% penicillin-streptomycin and grown to a monolayer at 37°C at 24 h. Before co-culture with lactobacilli, the culture medium was replaced with fresh medium without antibiotics. To determine the adhesion of lactobacilli, the cells were infected with a multiplicity of infection (MOI) of 100 of the lactobacilli and incubated at 37°C for 1.5 h. The bacteria were stained with Giemsa for 60 min to be observed under a light microscope (Eclipse TS100/Nikon Instrument Inc., Japan) with a camera (TrueChrome 4K/Fuzhou Tucsen Photonics Co., Ltd, Taiwan) at 1,000 magnification. A minimum of five randomly selected fields of view from each well were used for this analysis.

**Exopolysaccharide extraction**

Lactobacilli were inoculated in 25 mL MRS broth containing 2% sucrose at 37^o^C for 24 h. Bacterial cultures were centrifuged at 3,011 xg for 30 min to remove cells and their debris. EPS were precipitated from supernatants by adding 4 volumes of 95% ethanol, and the mixture was incubated at 4°C for 24 h [2]. After ethanol precipitation, the samples were centrifugated at 3,011 xg for 30 min, and the supernatant was removed. The precipitate of pure EPS was dried in the oven at 60°C for 24 h. The precipitates were re-suspended in distilled water, filtered 0.45 µm diameter filter, and stored at -80°C until further testing. The production of EPS was analyzed by the phenol-sulfuric method using glucose as a reference standard [3]. Exopolysaccharide production assay was conducted in biological triplicate to ensure reproducibility.

**Anti-*H. pylori* activity of EPS derived from lactobacilli**

*H. pylori* suspensions were adjusted to OD_600_=1 in F12 cell medium without FBS and antibiotic and were incubated under cell culture conditions at 37°C with a final concentration of EPS 500 ng/mL for 2 h. F12 medium was used as the control. The viability of *H. pylori* after 72 h co-incubation with EPS was evaluated by determining the viable bacterial count on Brucella agar containing 10% horse serum plates after incubation at 37°C under microaerophilic conditions. *H. pylori* survival rate (%) = [A1(Log CFU/mL)/A0(Log CFU/mL)] × 100, where A0 is the viable count of *H. pylori* at 0 h and A1 is the viable count of *H. pylori* after 2 h co-incubation with EPS. Anti-*H. pylori* activity assay was conducted in biological triplicate to ensure reproducibility.

**Figure S1.**

**(A).**


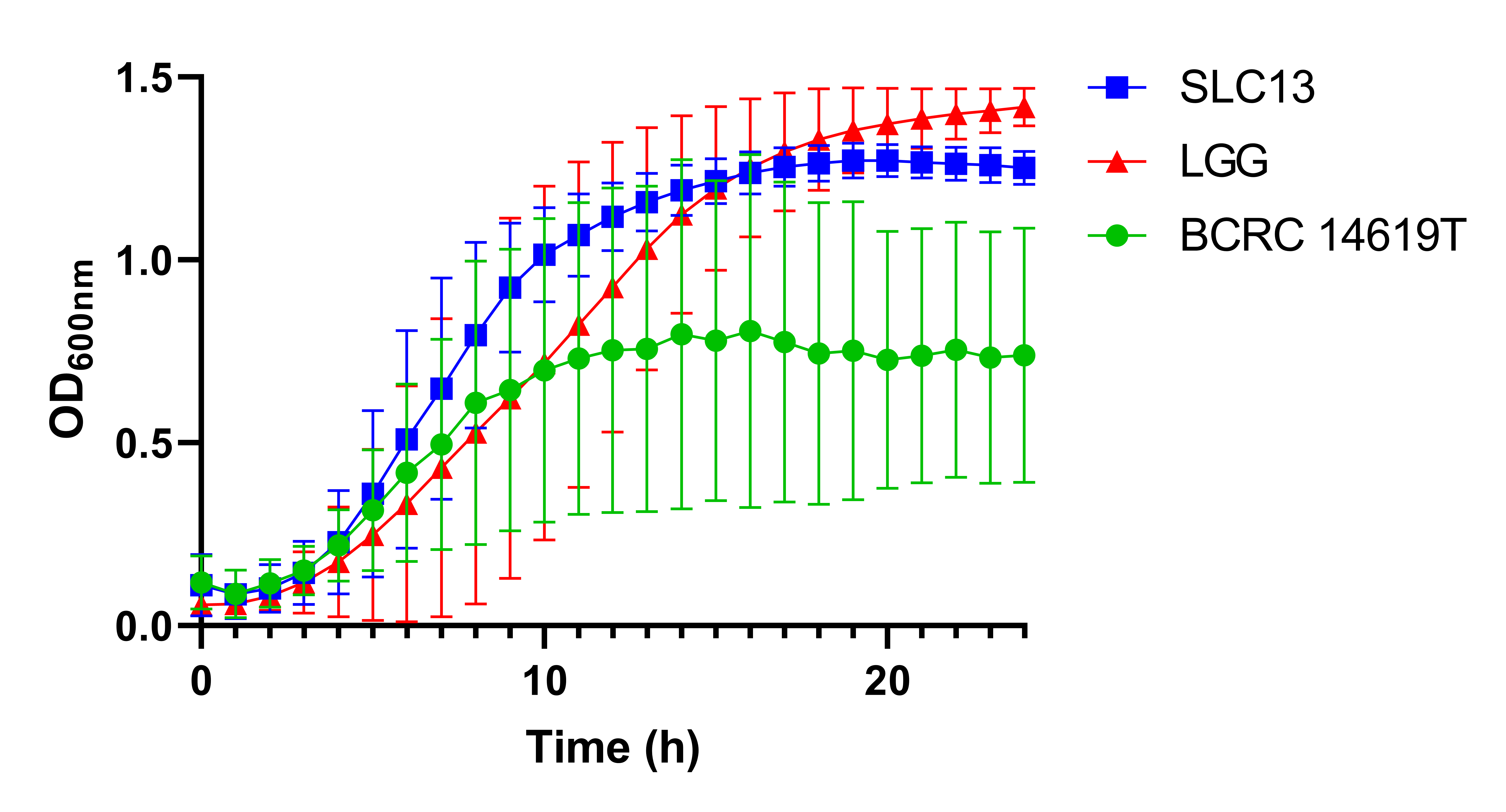


**(B).**


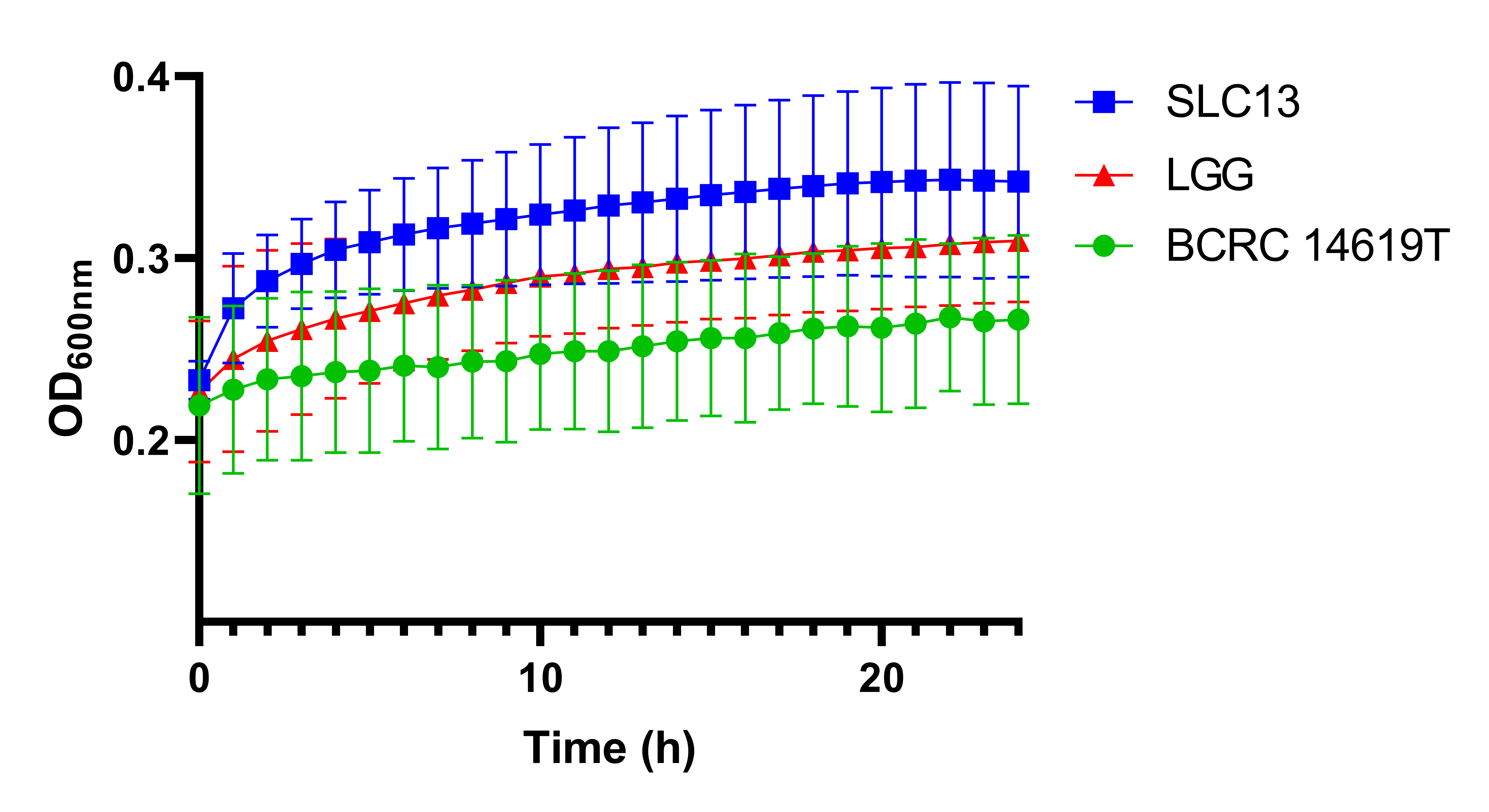


**(C).**


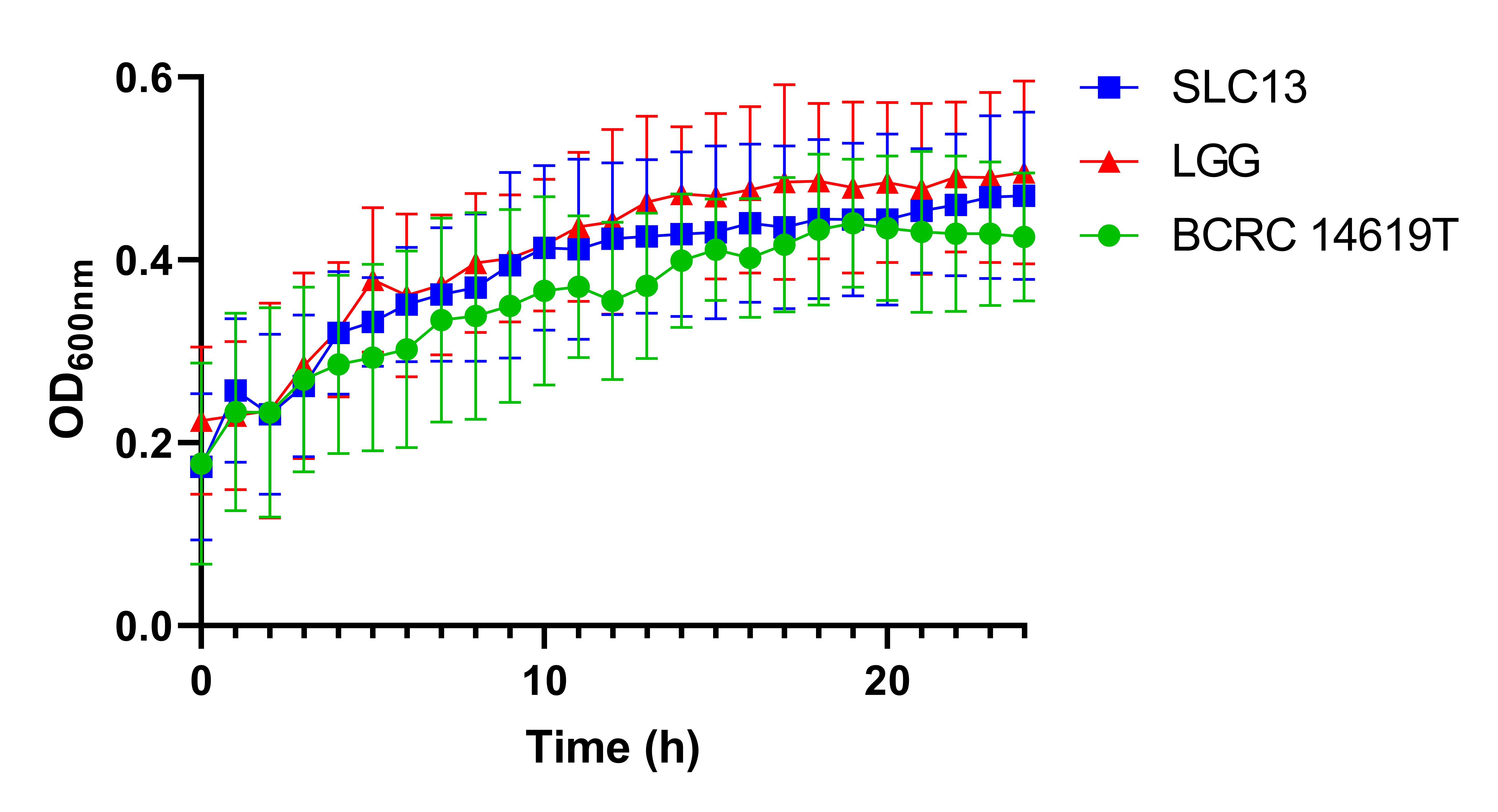


**Figure S1.** Growth curves of SLC13, LGG, and BCRC 14619T incubated for 24 h in MRS broth (pH 6.5) **(A)**, acidic MRS broth (pH 3.0) **(B)**, and MRS broth with 3% bile salts (pH 6.5) **(C)**. Error bars represent the standard deviation of biological triplicates.

**Figure S2.**


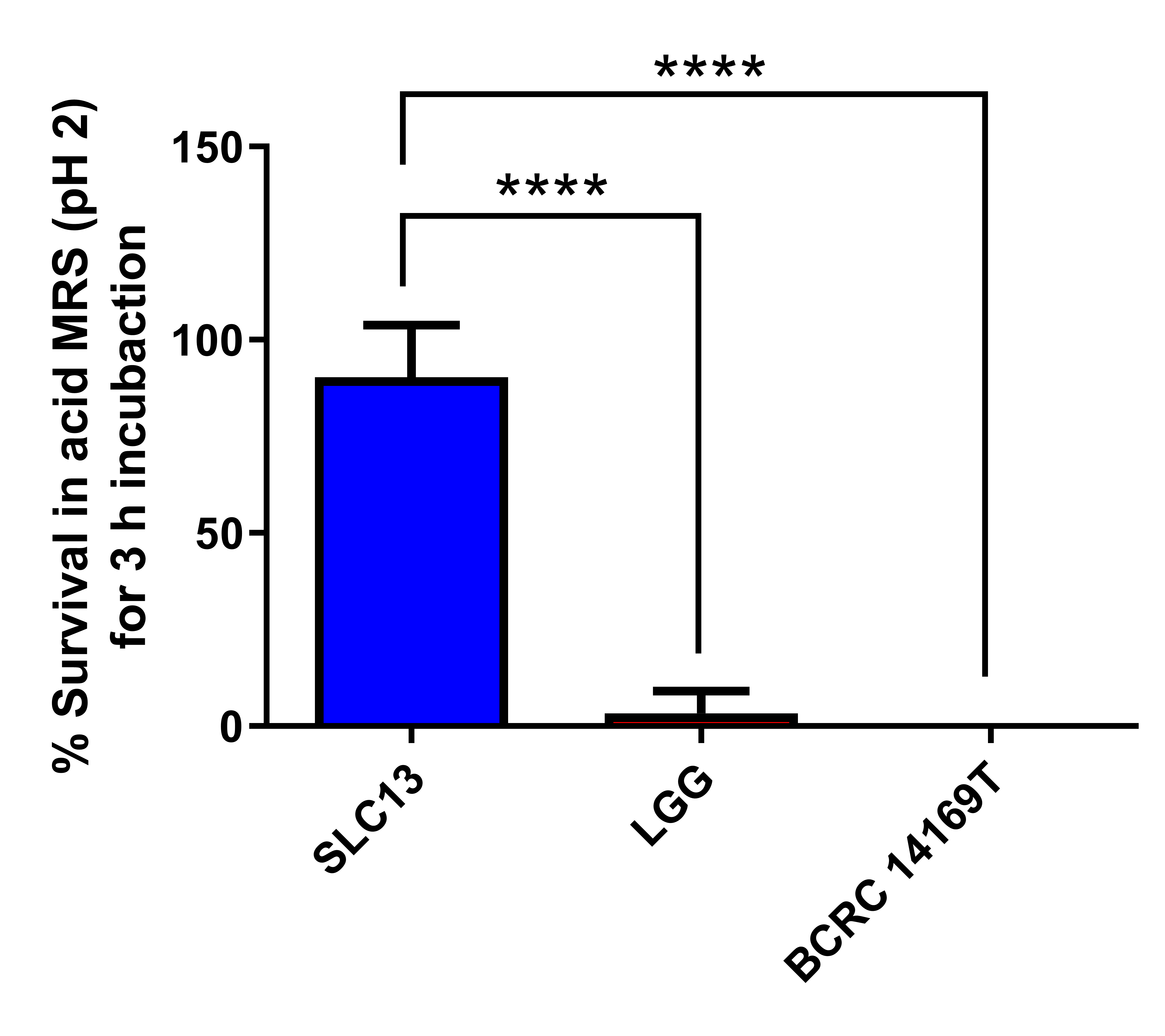


**Figure S2.** Survival of SLC13, LGG, and BCRC 14619T was determined by counting the viable cells after 3 hours incubation in MRS broth (pH 2.0). Error bars represent the standard deviation of biological triplicates. The survival rate of LGG and BCRC 14169T in acid MRS after 3 hours incubation was compared to SLC13 in acid MRS after 3 hours incubation. ****, *p* < 0.0001.

**Figure S3.**


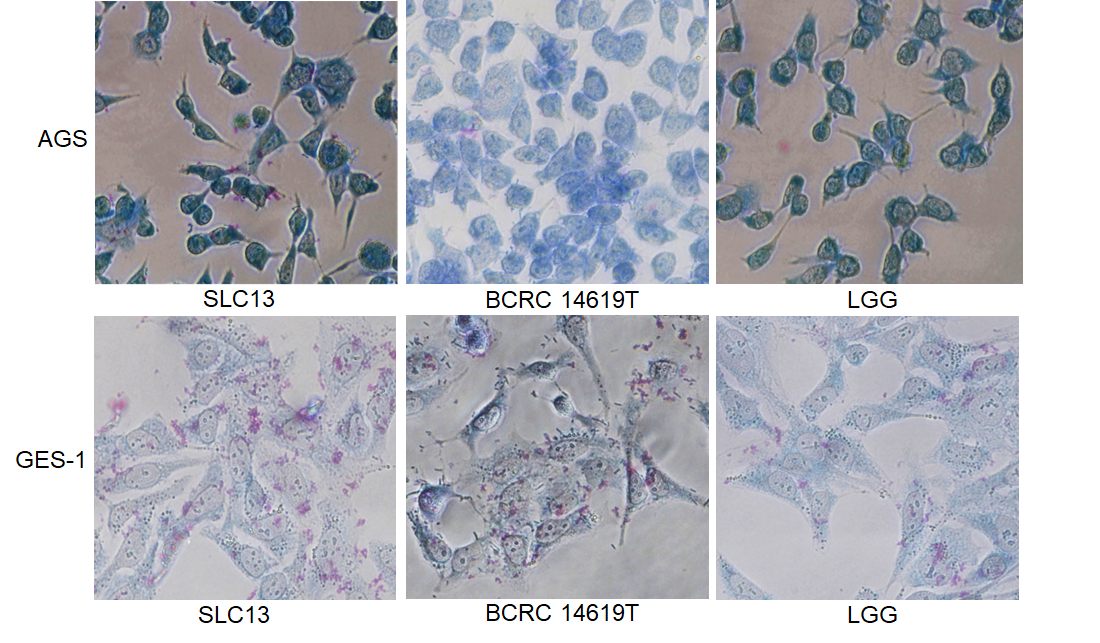


**Figure S3. Adhesion of SLC13, LGG, and BCRC 14619T to AGS and GES-1 cells.** The microscope observation of attachment of lactobacilli to AGS and GES-1 cells. The bacteria were stained with Giemsa and thus showed purple color.

**Figure S4.**

**Figure S4. Extraction of lactobacilli exopolysaccharide.** Exopolysaccharide production in the culture medium of lactobacilli SLC13, LGG, and BCRC 14619T in MRS broth containing 2% sucrose at 37^o^C for 24 h. Phenol-sulfuric acid method was used to measure the content of EPS using glucose as standard. EPS content was calculated according to the regression equation based on the standard curve, and then converted with dilution ratio. EPS, exopolysaccharide**.** NC, negative control (detection background of phenol-sulfuric mixture). Error bars represent the standard deviation of biological triplicates. ****, *p* <0.0001.

**Figure S5.**


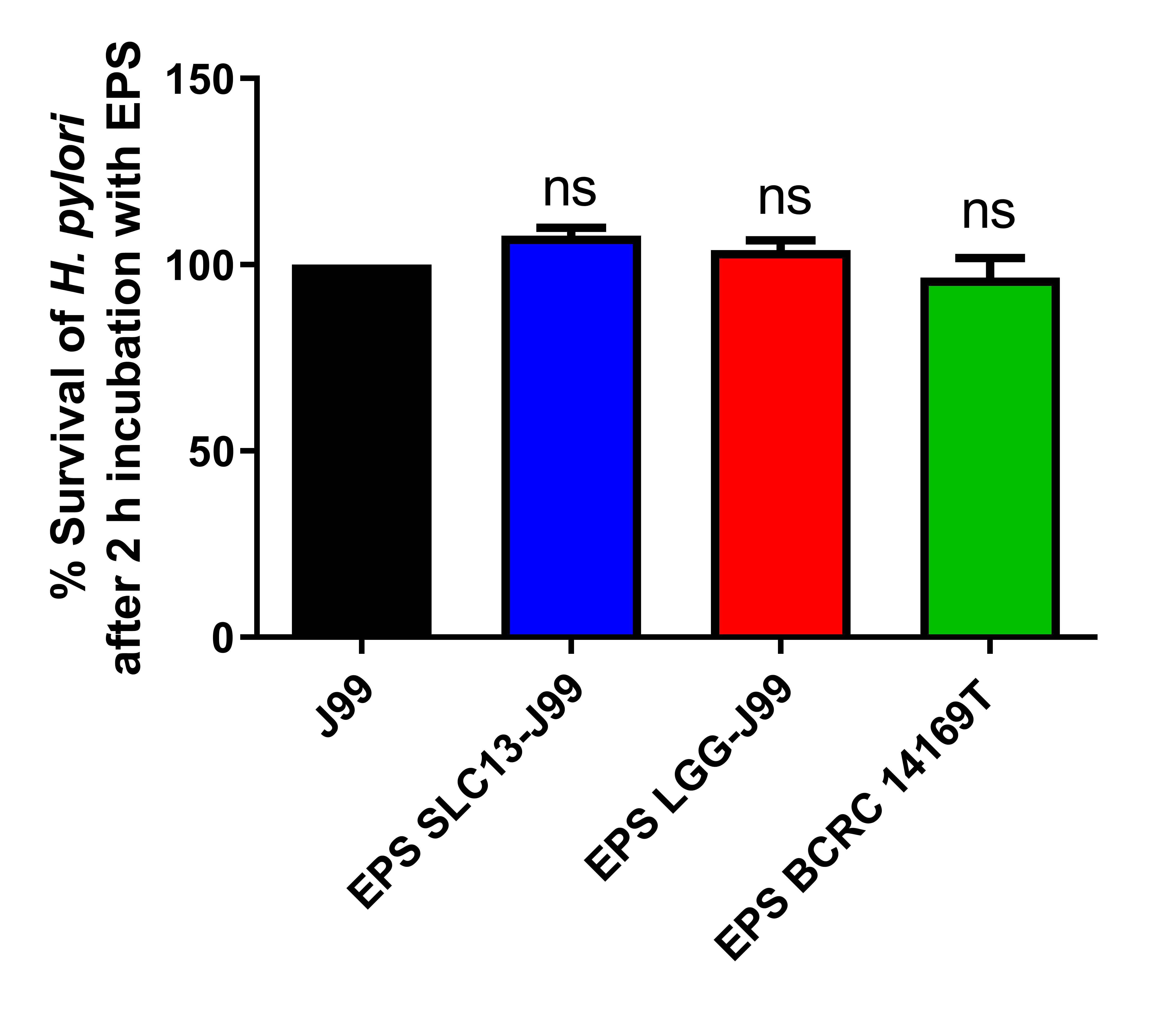


**Figure S5. Anti-*H. pylori* activity of lactobacilli** **exopolysaccharide.** The survival rate of *H. pylori* in the presence of 500 ng/mL EPS extracted from lactobacillus SLC13, LGG, and BCRC 14619T at 37^o^C for 2 h. EPS, exopolysaccharide. Error bars represent the standard deviation of biological triplicates. J99 alone without ESP treatment was used as a negative control. ns: no significant difference.

**References**

1. Huang JY, Kao CY, Liu WS, Fang TJ: Characterization of high exopolysaccharide-producing Lactobacillus strains isolated from mustard pickles for potential probiotic applications. *Int Microbiol* 2017, 20(2):75-84.

2. Chun-lei Z, Jia-qi L, Hai-tao G, Jie W, Ri-hua XJMčzupipm: Selection of exopolysaccharide-producing lactic acid bacteria isolates from Inner Mongolian traditional yoghurt. 2014, 64(4):254-260.

3. Torino M, Taranto M, Sesma F, De Valdez GFJJoAM: Heterofermentative pattern and exopolysaccharide production by Lactobacillus helveticus ATCC 15807 in response to environmental pH. 2001, 91(5):846-852.
